# Characterization of the genetic basis of local adaptation of wheat landraces from Iran and Pakistan using genome‐wide association study

**DOI:** 10.1002/tpg2.20096

**Published:** 2021-07-18

**Authors:** Uzma Hanif, Hadi Alipour, Alvina Gul, Li Jing, Reza Darvishzadeh, Rabia Amir, Faiza Munir, Muhammad Kashif Ilyas, Abdul Ghafoor, Sadar Uddin Siddiqui, Paul St. Amand, Amy Bernado, Guihua Bai, Kai Sonder, Awais Rasheed, Zhonghu He, Huihui Li

**Affiliations:** ^1^ Atta‐ur‐Rahman School of Applied Biosciences National Univ. of Sciences and Technology Islamabad Pakistan; ^2^ Dep. of Plant Production and Genetics, Faculty of Agriculture and Natural Resources Urmia Univ. Urmia Iran; ^3^ Institute of Crop Sciences Chinese Academy of Agricultural Sciences (CAAS), & CIMMYT‐China office 12 Zhongguancun South St. Beijing 100081 China; ^4^ Plant Genetic Resource Program, Bioresource Conservation Institute National Agricultural Research Center Islamabad 44000 Pakistan; ^5^ USDA Hard Winter Wheat Genetics Research Unit Manhattan KS 66506 USA; ^6^ International Wheat and Maize Improvement Center (CIMMYT) Texcoco Mexico; ^7^ Dep. of Plant Sciences Quaid‐i‐Azam Univ. Islamabad 45320 Pakistan

## Abstract

Characterization of genomic regions underlying adaptation of landraces can reveal a quantitative genetics framework for local wheat (*Triticum aestivum* L.) adaptability. A collection of 512 wheat landraces from the eastern edge of the Fertile Crescent in Iran and Pakistan were genotyped using genome‐wide single nucleotide polymorphism markers generated by genotyping‐by‐sequencing. The minor allele frequency (MAF) and the heterozygosity (*H*) of Pakistani wheat landraces (MAF = 0.19, *H* = 0.008) were slightly higher than the Iranian wheat landraces (MAF = 0.17, *H* = 0.005), indicating that Pakistani landraces were slightly more genetically diverse. Population structure analysis clearly separated the Pakistani landraces from Iranian landraces, which indicates two separate adaptability trajectories. The large‐scale agro‐climatic data of seven variables were quite dissimilar between Iran and Pakistan as revealed by the correlation coefficients. Genome‐wide association study identified 91 and 58 loci using agroclimatic data, which likely underpin local adaptability of the wheat landraces from Iran and Pakistan, respectively. Selective sweep analysis identified significant hits on chromosomes 4A, 4B, 6B, 7B, 2D, and 6D, which were colocalized with the loci associated with local adaptability and with some known genes related to flowering time and grain size. This study provides insight into the genetic diversity with emphasis on the genetic architecture of loci involved in adaptation to local environments, which has breeding implications.

## Core Ideas

- Genetic loci underpinning adaptability to the target environment were revealed by GWAS.
- Genes relate to flowering time and grain size were found to be associated with adaptability.
- The use of historical agro‐climatic data was useful in revealing loci that affect wheat adaptability.

**Abbreviations**
ACV: agro‐climatic variables
EigenGWAS: eigenvector decomposition GWAS
EnvGWAS: environmental GWAS
FarmCPU: fixed and random model circulating probability unification
Fst: fixation index
GBS: genotyping‐by‐sequencing
GWAS: genome‐wide association study
LD: linkage disequilibrium
LOESS: locally weighted polynomial regression
MAF: minor allele frequency
PCA: principal component analysis
PCR: polymerase chain reaction
PIC: polymorphic information content
Q–Q: quantile–quantile
QTL: quantitative trait loci
SNP: single nucleotide polymorphism
Ts: transition type
Tv: transversion type

## 1 INTRODUCTION

The bread wheat (*Triticum aestivum* L.) genome is highly diversified with several distinct classes based on vernalization requirements, growth habit, planting times, and grain quality (Salamini et al., 2002). The genetic diversity of cultivated wheat has critically decreased due to the modern breeding activities that focus on a few major developmental traits (Reif et al., 2005). Of the four cultivated species (bread wheat, einkorn [*Triticum monococcum* Boiss.], emmer [*Triticum turgidum* ssp. *dicoccum* (Schrank) Schübl.], and spelt [*Triticum spelta* L.]), 95% of the cultivation and consumption is bread wheat. Modern bread wheat cultivars have reduced genetic variation with only 30% of the genetic variation found in their wild relatives (Reif et al., 2005). The loss of diversity in hexaploid wheat is mainly due to the reproductive isolation between closely related species of different ploidy levels because some of the wild relatives cannot make fertile hybrids upon hybridization (Mujeeb‐Kazi et al., 2013). The reduction in genetic diversity is also attributed to the continuous artificial selection during domestication and modern wheat breeding, which positively selected genomic regions that influence productivity traits (Cavanagh et al., 2013; Jordan et al., 2015; Zhou et al., 2018). Landraces are traditional wheat varieties grown in farmers’ fields before modern breeding was established and thus have unexplored genetic variations with unused alleles for tolerance to both biotic and abiotic stresses, end‐use quality, and high resource use efficiency (Dwivedi et al., 2016; Lopes et al., 2015). Wheat landraces have the advantage over other wheat wild relatives of being more easily crossed with cultivated bread wheat. The large collections of landraces stored in gene banks have been characterized using modern genomic tools to systematically use beneficial traits in wheat breeding (Riaz et al., 2017; Sehgal et al., 2015; Vikram et al., 2016; Winfield et al., 2017).

Bread wheat evolved in the “Fertile Crescent” and then gradually spread to the rest of the world. N.I. Vavilov placed the origin of bread wheat to a region spanning an area from Afghanistan and Turkmenistan to Transcaucasia (Vavilov, 1926; Vavilov, 1992). The molecular evidence suggested the center of origin to be Transcaucasia and northwestern Iran (Dvorak et al., 1998). Scientific and archeological evidence suggested that Iran and Pakistan are important countries with reference to the center of diversity for cereals (Kihara et al., 1965). The southwestern Iran and Mehrgarh civilization in southwestern Pakistan are considered to be to be one of the main centers of diversity for bread wheat (Dvorak et al., 2011), barley (*Hordeum vulgare* L.), and einkorn wheat (Fuller, 2006; Morrell & Clegg, 2007). Thus, the in‐depth diversity analyses of landraces from these geographic regions may highlight selection patterns in response to local environments and explain the polygenic adaptation.

Progress has been made in whole genome sequencing of bread wheat and its immediate progenitors (IWGSC, 2018; Rasheed & Xia, 2019), which has resulted in significant achievements in genomic studies in understanding the genetic diversity and improved adaptation of beneficial traits in bread wheat and aids the discovery of new genes for beneficial traits (Rasheed & Xia, 2019 ). In this study, we resequenced a panel of 512 landraces from Iran and Pakistan using genotyping‐by‐sequencing (GBS) to identify the genetic regions that were selected for adaptation during wheat domestication and early breeding events, and to evaluate the genetic diversity in one of the major wheat diversity centers. In addition, we used geo‐referenced agro‐climatic attributes from the landrace collection sites as proxy traits and conducted genome‐wide association studies (GWAS) to identify the genomic regions associated with bread wheat local adaptability.

## 2 MATERIALS AND METHODS

### 2.1 Genetic materials

A panel of 512 wheat landraces was used in this study. This includes 251 landraces, mainly from Pakistan, collected from 1931 to 1968 that were deposited at the Plant Genetic Resource Institute, National Agriculture Research Center, Islamabad, Pakistan (Supplemental Table S1), and 261 Iranian landraces that were collected from different climate zones between 1931 and 1968 and deposited in the USDA‐ARS National Plant Germplasm System, Aberdeen, ID (Supplemental Table S2).

### 2.2 DNA extraction and GBS

Genomic DNA was extracted using a modified cetyltrimethyl ammonium bromide method (Saghai‐Maroof et al., 1984) from five 2‐wk‐old seedlings of each sample. DNA concentration was quantified using the Quant‐iT PicoGreen dsDNA Assay (Life Technologies Inc.) and normalized to 20 ng μl^‐1^ for library construction. The GBS libraries were constructed following Poland et al. (2012). In brief, genomic DNA was digested using the restriction enzymes *Pst*I and *Msp*I (New England BioLabs Inc.), and barcoded adapters were ligated to each DNA sample using T4 ligase (New England BioLabs Inc.). All of the ligated products from each plate were pooled and cleaned‐up using the QIAquick PCR Purification Kit (Qiagen Inc.). Primers complementary to both adaptors were used for polymerase chain reaction (PCR). The PCR products were then cleaned up again using the QIAquick PCR Purification Kit and quantified using the Bioanalyzer 7500 Agilent DNA Chip (Agilent Technologies, Inc.). After size selection for 250–300 bp fragments in an E‐gel system (Life Technologies Inc.), the concentration of each library was estimated by the Qubit 2.0 fluorometer using Qubit dsDNA HS Assay Kit (Life Technologies Inc.). The size‐selected library was sequenced on an Ion Proton sequencer (Life Technologies Inc.).

First, sequence reads were trimmed to 64 bp, and then identical reads were grouped into sequence tags. The unique tags were aligned internally allowing mismatches of up to 3 bp to identify single nucleotide polymorphisms (SNPs) within the tags. Single nucleotide polymorphism calling was conducted using the Universal Network Enabled Analysis Kit GBS pipeline (Lu et al., 2013), which is part of the TASSEL 4.0 bioinformatics analysis package (Bradbury et al., 2007). Reads with a low quality score (<15) were removed, and SNPs with heterozygosity <10%, a minor allele frequency (MAF) >1%, and missing data <20% were used for further analysis. Sequence reads were aligned to the International Wheat Genome Sequencing Consortium (IWGSC) reference genome sequence (Version 1.0) (IWGSC, 2018) using BLASTn.

### 2.3 Data analyses

The basic statistics for the allele frequency, heterozygosity and genetic diversity were performed using TASSEL (Version 5.0). A diversity tree was built using the Neighbor‐Joining algorithm (Saitou & Nei, 1987) that relaxes the assumption of equal mutation rates over space and time and produces an unrooted tree. The confidence interval of the genetic relationships among the accessions was determined by performing 1,000 bootstraps with the results expressed as percentages at the main nodes of each branch. For population structure, a model‐based Bayesian cluster analysis was performed using STRUCTURE (Version 2.3.4) (Pritchard et al., 2000). The structure analysis was run 10 times for each *K* value (probability of best fit into each number of assumed clusters) (*K* = 1–8) using a burn‐in period of 10,000 steps and 10,000 Monte Carlo steps and an admixture model. All parameters were set to default values recommended by the manufacturer (Pritchard et al., 2010). *K* was estimated by an ad hoc statistic Δ*K* based on the rate of change in the log probability of data between consecutive *K* values (Evanno et al., 2005).

The squared correlation of allele frequency (*r* ^2^) was calculated using PLINK 1.9 (https://www.cog‐genomics.org/plink2; Chang et al., 2015) to evaluate the linkage disequilibrium (LD) in both sets of landraces independently and combined. The pairwise *r* ^2^ values were plotted against genetic distance in a 1‐kb window, and a locally weighted polynomial regression (LOESS) curve was fitted using R software. The pattern of LD decay was determined as the genetic distance where LOESS curve intercepts the *r* ^2^ threshold of 0.1.

**Core Ideas**

- Genetic loci underpinning adaptability to the target environment were revealed by GWAS.
- Genes relate to flowering time and grain size were found to be associated with adaptability.
- The use of historical agro‐climatic data was useful in revealing loci that affect wheat adaptability.

The eigenvector decomposition GWAS (EigenGWAS), which uses genome‐wide association studies of eigenvectors, was used to identify the loci under selection (Chen et al., 2016). We used SNPs that fulfils the quality control criteria and generated a genetic relationship matrix to calculate the top 10 eigenvalues and their corresponding eigenvectors. The effect of SNP was calculated by regressing each SNP on a selected eigenvector. In principle, the estimated genetic effect for each locus is driven by genetic drift and selection, which are random. To remove genetic drift effects, we adjusted the *p*‐value with genomic control (GC) factor, and consequently the corrected *p*‐value, *P*_GC_, was used to detect loci under selection. The significance threshold for loci under selection was determined by reshuffling the first eigenvector 1,000 times, and the 95th quantile of the most significant *p*‐values from the 1,000 permutations was selected as the significance threshold. In this study, the −log_10_ (*p*‐value) of 5.87, which is equivalent to an experiment‐wise type I error rate of .05, was used as the significance threshold for the EigenGWAS analyses on the 10 eigenvectors.

### 2.4 GIS data extraction and environmental GWAS

The georeferenced longitude and latitude positions of landrace collection sites were retrieved from the passport information of landraces from Pakistan and Iran. The landraces from other countries (*n* = 24) were excluded from the analysis. Climate data were extracted from extrapolated climate grids (Fick & Hijmans 2017) at a 30‐s (∼1 km) resolution. Raw data files for monthly long‐term (1970–2000) minimum (*T*_min_), maximum (*T*_max_), average (*T*_avg_) temperatures, rainfall (PREC), vapor pressure (VAPR), solar radiation (SRAD), and soil pH (ph5) were downloaded and converted to Environmental System Research Institute (ESRI) grid format for storage and extraction (Hengl et al., 2017). Georeferenced locations for accessions were checked for errors and then used to extract climate values for each location using the spatial analyst toolbox in ESRI ArcMap 10.6 (Environmental System Research Institute, 2018).

The GWAS was conducted using a recently developed model selection algorithm, the fixed and random model circulating probability unification (FarmCPU) (Liu et al., 2016) with eight GIS data (*T*_max_, *T*_min_, *T*_avg_, SRAD, VAPR, latitude, longitude, and PREC) as response variables. The FarmCPU takes into account the confounding problem between covariates and test markers using a fixed effect model (FEM) and a random effect model (REM). The first five principal components calculated using TASSEL were used as covariates. The default *p*‐value threshold that FarmCPU used was Bonferroni‐corrected threshold of .01. The Bonferroni‐corrected threshold was overly restrictive when the LD among genotypic markers was large, so the threshold was calculated using the formula *p* = .05/number of markers using 1,000 permutations. In this function, the phenotypes were permuted to break the relationship with the genotypes. A vector of minimum *p*‐values of each experiment was output and the 95% quantile value of the vector was recommended for p.threshold in the FarmCPU model. The threshold calculated by FarmCPU for the given traits was 4.68, which was used as a threshold to define marker‐trait associations (MTAs). The quantile–quantile (Q–Q) plot was used for assessing fitness of the model to population structure.

## 3 RESULTS

### 3.1 Genetic diversity

**FIGURE 1:**
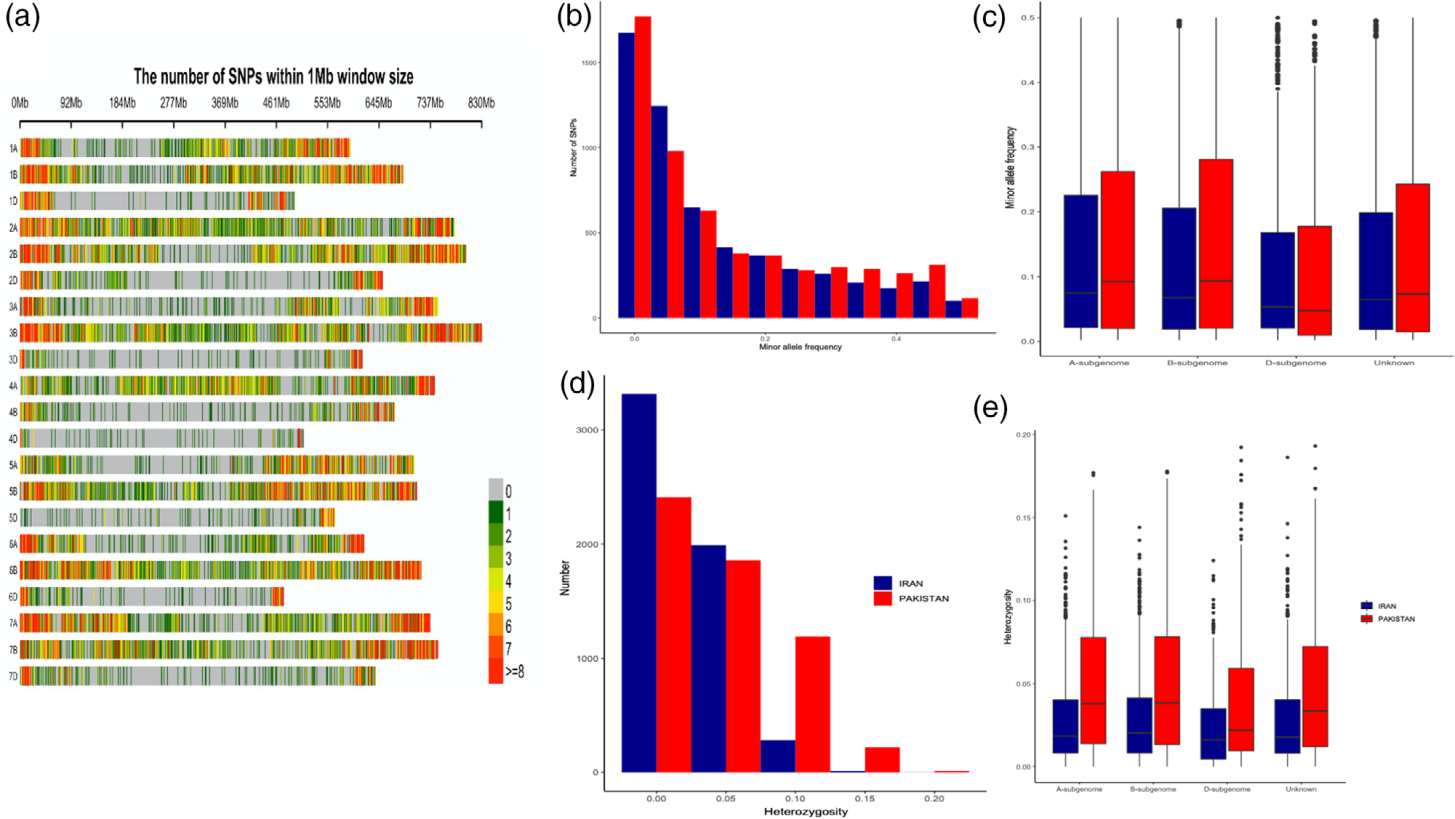
(a) Summary statistics visualization of single nucleotide polymorphisms (SNPs) in landraces from Iran and Pakistan; SNP density plot of all wheat chromosomes in terms of number of SNPs and Mb window. Histograms showing (b) minor allele frequency and (c) heterozygosity and box‐plot showing distribution of (d) minor allele frequency and (e) heterozygosity on the A, B, and D‐genomes separately for landraces from Iran (blue) and Pakistan (red)

A total of 43,868 SNPs with <80% missing data were identified in the panel of 512 Pakistani and Iranian wheat landraces, and 33,961 (∼77.4%) of them were mapped to the IWGSC wheat reference genome (Version 1.0) (IWGSC, 2018). The highest number of SNPs were mapped on the B genome (17,186), followed by the A genome (13,012) and the D genome (3,763) (Figure 1a). The B genome had the most transition‐type (Ts) SNPs (1,691), and the D genome had the least (681) (Supplemental Tables S3 and S4). A similar chromosome distribution pattern was observed for transversion‐type (Tv) SNPs; however, Tv SNPs were much more abundant (64.3%) than Ts SNPs (35.7%) with a Ts/Tv SNP ratio of 1.80. The Ts/Tv ratios in the A and B genomes were significantly higher than those in the D genome. As expected, more A/G and C/T transitions were observed than G/A and T/C transitions. On the other hand, the frequencies of C/G, A/C, G/T, and A/T transversions were higher than G/C, C/A, T/G, and T/A transversions (Supplemental Tables S3 and S4).

More monomorphic SNPs (997) were observed in Pakistani landraces than in Iranian landraces (543), and these monomorphic SNPs were removed for subsequent further SNP analyses except in EigenGWAS for selective sweeps. Average gene diversity and polymorphic information content (PIC) values were slightly higher in Pakistani landraces (0.20 and 0.17) than those in Iranian landraces (0.18 and 0.15), respectively (Table 1). Similarly, the level of heterozygous SNPs was higher (8.41%) in Pakistani landraces than in Iranian landraces (5.88%). In general, the mean values of MAF and heterozygosity (*H*) were slightly higher for the Pakistani wheat landraces (MAF = 0.19, *H* = 0.08) than these for Iranian landraces (MAF = 0.17, *H* = 0.05) (Table 1; Figure 1b–e).

**TABLE 1:** Summary of allelic diversity for Pakistani and Iranian wheat landraces

	Sample size	No. of polymorphic SNPs	Gene diversity	Heterozygosity	Polymorphic information content
Pakistani	251	42,871	0.202	0.084	0.17
Iranian	261	43,325	0.184	0.059	0.15
Total	512	44,255	0.194	0.033	0.16

*Note*. SNP, single nucleotide polymorphism.

**TABLE 2:** Linkage disequilibrium patterns of the single nucleotide polymorphism markers in the wheat chromosomes and genomes of 251 Pakistani and 261 Iranian landraces anchored to the International Wheat Genome Sequencing Consortium wheat reference genome (Version 1.0) (IWGSC, 2018)

	Iran				Pakistan			
Chromosome	Total	*p *< .01	Distance	*r* ^2^	Total	*p *< .01	Distance	*r* ^2^
			Mb				Mb	
1A	8,514	1,829 (21.48%)	8.78	.059	7,947	3,042 (38.28%)	9.15	.100
1B	9,576	2,627 (27.43%)	6.63	.065	8,768	2,700 (30.79%)	7.51	.094
1D	3,807	771 (20.25%)	20.89	.05	2,892	805 (27.84%)	20.58	.093
2A	9,110	2,139 (23.48%)	7.43	.06	7,521	2,259 (30.04%)	7.84	.077
2B	12,672	3,102 (24.48%)	6.10	.064	11,778	4,288 (36.41%)	5.70	.096
2D	6,024	909 (15.09%)	11.41	.076	3,098	797 (25.73%)	10.70	.113
3A	4,927	963 (19.55%)	11.04	.055	9,815	2,380 (24.25%)	10.83	.071
3B	11,804	2,789 (23.63%)	6.27	.060	10,614	2,995 (28.22%)	6.45	.080
3D	4,011	642 (16.01%)	16.56	.062	2,694	671 (24.91%)	16.32	.095
4A	5,916	1,076 (18.19%)	10.55	.07	6,058	1,558 (25.72%)	11.06	.089
4B	2,807	330 (11.76%)	11.49	.040	2,999	727 (24.24%)	9.72	.071
4D	641	114 (17.78%)	22.08	.052	271	48 (17.71%)	19.31	.039
5A	5,420	1,114 (20.55%)	10.29	.047	5,491	1,336 (24.33%)	9.87	.067
5B	10,219	2,039 (19.95%)	8.74	.046	9,603	2,820 (29.37%)	9.18	.082
5D	2,343	276 (11.78%)	34.13	.031	1,198	201 (16.78%)	27.04	.051
6A	5,621	1,598 (28.43%)	11.97	.068	5,714	1,711 (29.94%)	10.54	.075
6B	10,006	1,973 (19.72%)	6.29	.045	8,340	2,328 (27.91%)	5.98	.087
6D	2,820	453 (16.06%)	25.39	.039	1,146	216 (18.85%)	27.15	.062
7A	10,162	2,104 (20.70%)	8.66	.055	11,115	2,812 (25.30%)	7.21	.077
7B	10,180	2,130 (20.92%)	6.04	.047	10,947	3,737 (34.14%)	6.03	.100
7D	4,498	605 (13.45%)	16.71	.045	2,128	347 (16.31%)	14.94	.067
A genome	49,670	10,823 (21.79%)	9.47	.0612	53,661	15,098 (28.14%)	9.31	.081
B genome	67,264	14,990 (22.29%)	6.85	.0559	63,049	19,595 (31.08%)	6.89	.090
D genome	24,144	3,770 (15.61%)	18.87	.0572	13,427	3,085 (22.98%)	17.67	.090
Total	141,078	29,583 (20.97%)	9.8	.0580	130,137	37,778 (29.03%)	9.00	.0867

### 3.2 Population structure and geographic and genetic diversity in the landrace panel

**FIGURE 2:**
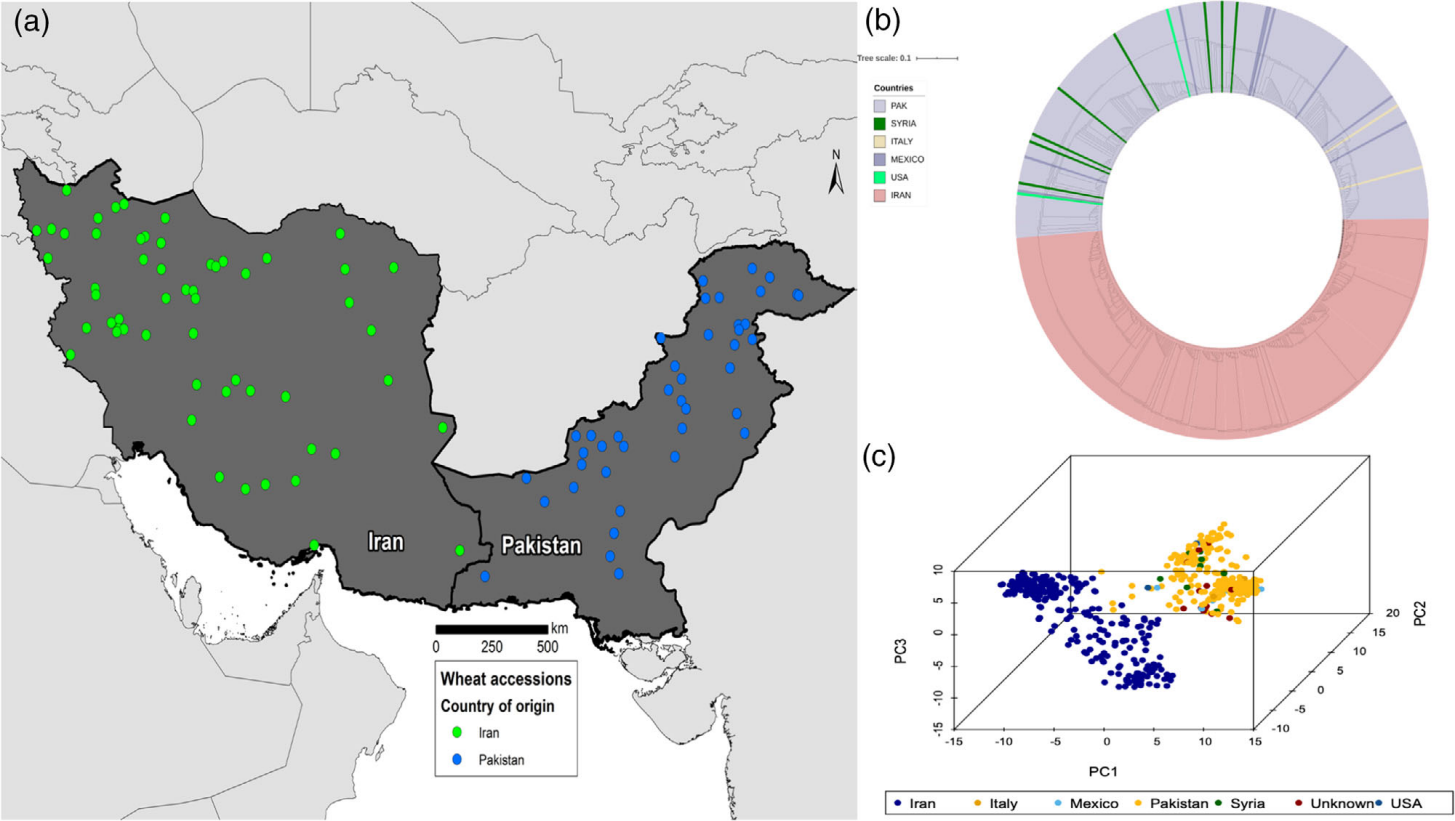
(a) Landrace distributions in Iran and Pakistan covering all the eco‐geographic regions in both countries. (b) Phylogenetic tress showing clustering of landraces used in this study. (c) Principal component (PC) analysis based on single nucleotide polymorphisms derived from genotyping‐by‐sequencing

**FIGURE 3:**
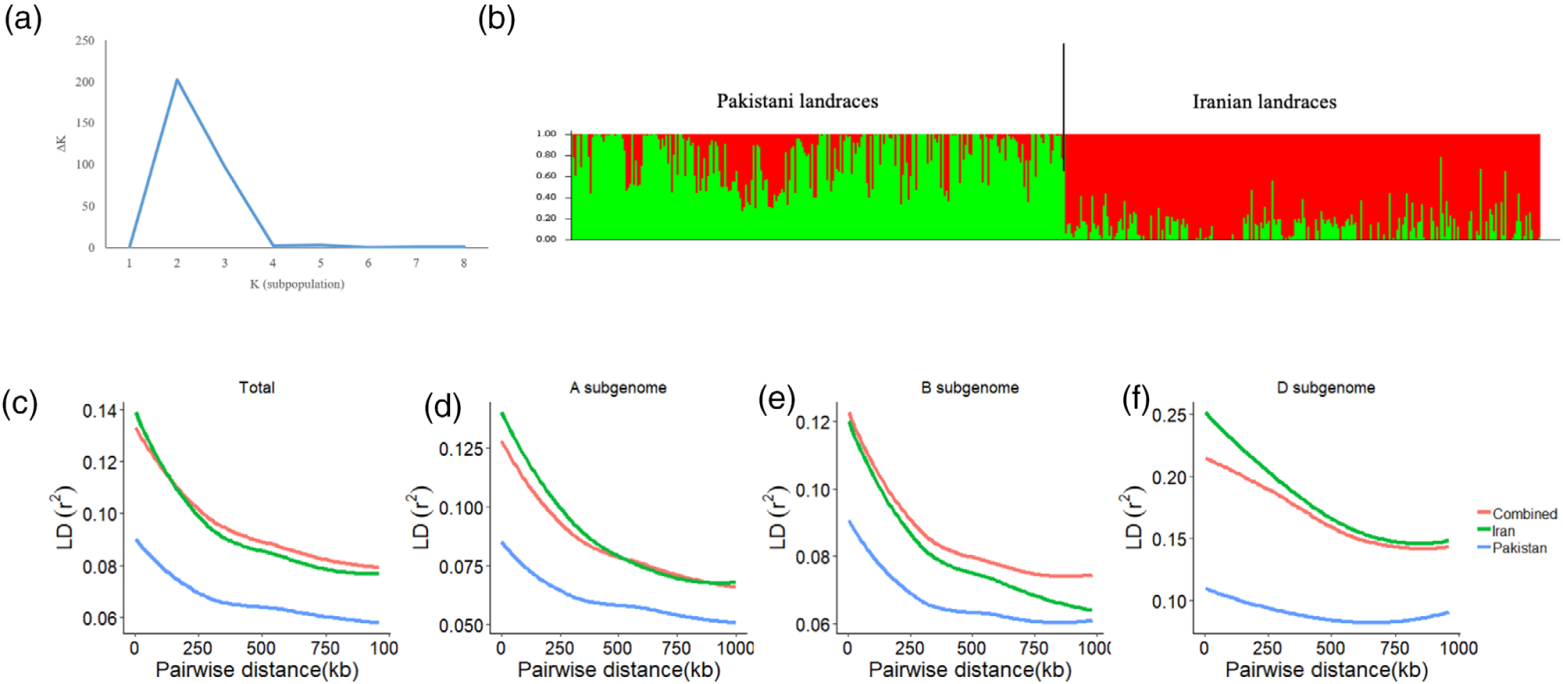
Population structure and linkage disequilibrium (LD) analyses in landraces from Iran and Pakistan. (a) Plot of the average logarithm of the probability of data Δ*K*, as second rate of change of the number of assumed subgroups (*K*), with *K* allowed to range from 2 to 20. (b) Membership coefficient where each horizontal line represents one wheat line and partitioned into two subpopulations. (c) Linkage disequilibrium decay based on genome‐wide markers from all wheat chromosomes, (d) A‐genome chromosomes, (e) B‐genome chromosomes, and (f) D‐genome chromosomes

Almost all Pakistani landraces were clearly separated from Iranian landraces, with three Pakistani landraces in close proximity to the Iranian subpopulation in the principal component analysis (PCA)‐ biplot (Figure 2a). Two of these landraces, accession 11186 and 11342, were collected from Balochistan (an area close to Iranian border) and one landrace (accession 12219) was from northern Pakistan. None of Iranian landraces was included in the Pakistani subpopulation. Cluster analysis using weighted pair group method analysis and principal coordinate analysis also classified the panel into two subpopulations in accordance with population structure analysis (Figure 2b and c, respectively). The Pakistani landraces were distributed over two major clusters, and the Iranian landraces were distributed over 10 independent clusters. Population structure of the landrace panel was estimated using STRUCTURE software based on genome‐wide SNP markers. The highest Δ*K* value was observed at *K* = 2, suggesting two subpopulations in the panel (Figure 3a).

### 3.3 Analysis of molecular variance and LD

The analysis of molecular variance for the landrace panel using 6,229 SNPs showed a much greater variation within a population (77.41%) than between the populations (22.59%, *p* < .001). The higher value of fixation index (Fst) value (.23) between the Pakistani and Iranian subpopulations suggests high genetic differentiation between the two subpopulations. The highest genetic differentiation between Pakistani and Iranian subpopulations were observed on the B genome (Fst = .28).

### 3.4 Pairwise LD and LD decay in Pakistani and Iranian wheat landraces

All the SNPs mapped to the IWGSC wheat reference genome (Version 1.0) (IWGSC, 2018) were used to estimate the extent of LD for all the accessions in the landrace panel (Table 2). The LD decay plots in Figure 3b show distance in kilo‐base pair vs. LD (*r* ^2^). The LD decay between the Iranian and Pakistani subpopulations revealed higher *r* ^2^ values in Pakistani landraces (Figure 3c). The number of SNP pairs in the A and B genome (Figure 3d and e, respectively) was almost the same between the two subpopulations while the number of SNP pairs in the D genome was much higher in the Iranian subpopulation than in Pakistani subpopulation (Figure 3f). The average distance between SNP pairs was slightly higher (9.83 Mb) in the Iranian subpopulation than in the Pakistani subpopulation (9.0 Mb), with overall *r* ^2^ values of .058 and .086, respectively. The LD decayed to .2 among Iranian landraces at an average of 4.5 Mb distance, compared with 15.2 Mb in Pakistani landrcaes.

### 3.5 Agro‐climatic variables for Iran and Pakistan

The climatic data were extracted from extrapolated climate grids at 30 s (∼1 km) resolution for all landraces from Pakistan and Iran. Seven agro‐climatic variables (ACVs) were included in this study: longitude, latitude, *T*_max_, *T*_min_, PREC, VAPR, and SRAD. Pakistani wheat landraces were distributed between 70.62° E and 33.50° N with maximum values of 63.014–75.62° E, 26.15–36.16° N, whereas the Iranian wheat landraces were distributed 45°–62° E, 25.55–38.7° N. The means of other ACVs such as *T*_max_ and *T*_min_ were 26.19 °C and 11.16 °C for Pakistan compared with 25.55 °C and 0 °C for Iran (Table 3). Similarly, annual PREC was 427.04 mm, VAPR was 1.12 Pa, and SRAD was 17,651.53 W/m^2^ in Pakistan, whereas *T*_max_ and *T*_min_ were 26.2 °C and 11.16 °C, and annual PREC was 326.14 mm, VAPR was 0.74 Pa, and SRAD was 17,419.18 W/m^2^ in Iran (Table 3).

**TABLE 3:** Mean argo‐climatic variables (monthly, 1970–2000) collected at 30 s (∼1 km) resolution from the site of landraces collection from Iran and Pakistan

Agro‐climatic variables	Combined	Pakistan	Iran
Longitude			
Mean	59.62	70.62	50.85
Maximum	76.62	75.62	62
Minimum	45	63.01	45
Latitude			
Mean	33.5	32.36	34.4
Maximum	38.7	36.16	38.7
Minimum	25.55	26.15	25.55
*T*_max_			
Mean	23.58	26.19	21.5
Maximum	35.65	35.65	31.5
Minimum	0	11.84	0
*T_min_*			
Mean	9.19	11.16	7.63
Maximum	21.775	19.23	21.775
Minimum	0	0.8	0
PREC			
Mean	370.99	427.04	326.14
Maximum	1,429	1,429	1,028
Minimum	70	85	70
VAPR			
Mean	0.91	1.12	0.74
Maximum	2.33	1.94	2.33
Minimum	0.4	0.53	0.4
SRAD			
Mean	17,522.45	17,651.53	17,419.18
Maximum	20,160.5	20,046.92	20,160.5
Minimum	14,064	15,493.17	14,064

*Note. T*_min_, minimum temperature; *T*_max_, maximum temperature; PREC, rainfall; VAPR, vapor pressure; SRAD, solar radiation.

### 3.6 Correlation between ACVs of Iranian and Pakistani wheat landraces

**FIGURE 4:**
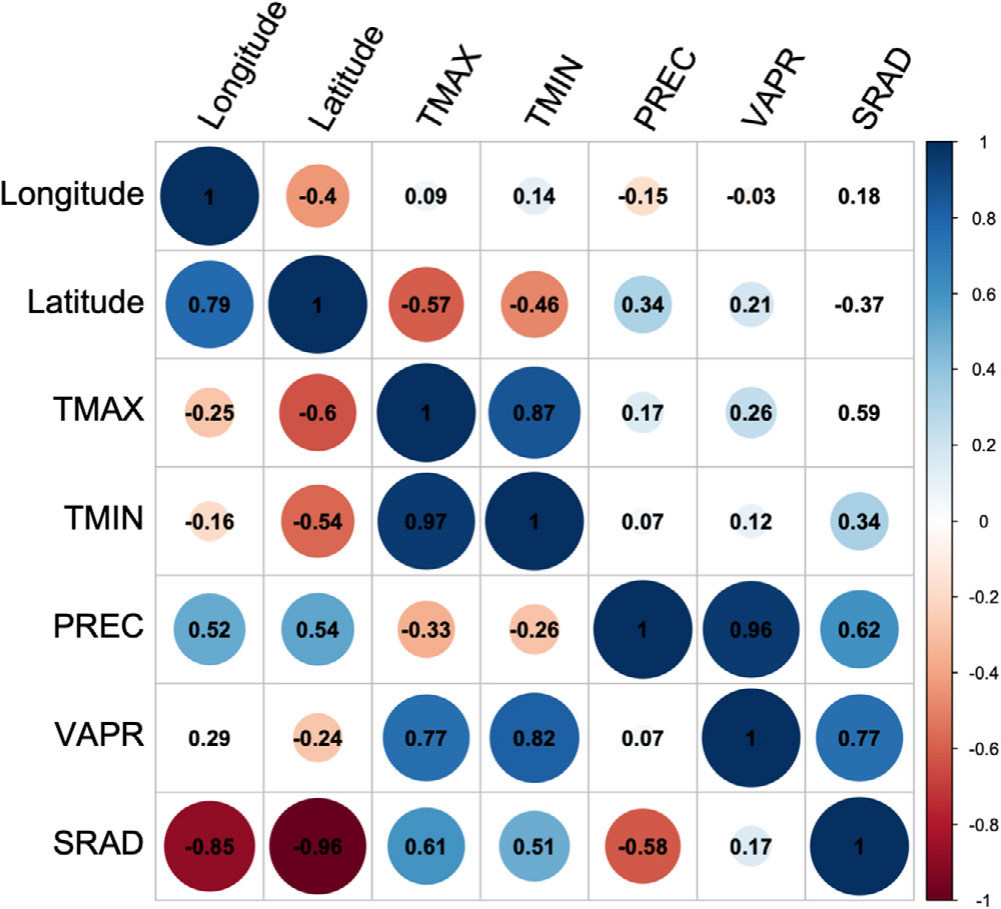
Coefficient of correlation between agro‐climatic variables collected from the landraces collection sites in Iran and Pakistan. Upper diagonal represents a correlation between agro‐climatic variables in Iran and lower diagonal represents a correlation between agro‐climatic variables in Pakistan. *T*_min_, minimum temperature; *T*_max_, maximum temperature; PREC, precipitation; VAPR, vapor pressure; SPAD, solar radiation

There was a positive correlation (*r* = .53) between annual precipitation (PREC) and longitude or latitude in Pakistan (Figure 4; Supplemental Table S5). Iran had a positive correlation between PREC and latitude (*r* = .33) but a negative correlation between PREC and longitude (*r* = −.14). A high positive correlation between PREC and VAPR was observed in Iran (*r* = .98), but not in Pakistan where *r* values were from .06 to −.4. The maximum (*T*_max_) and minimum (*T*_min_) temperatures had weak positive associations with PREC and VAPR, respectively, in Pakistan, but a highly positive correlation with VAPR and a negative correlation with PREC in Iran (Supplemental Table S5). The SRAD was negatively correlated with latitude, longitude, and PREC, but positively correlated with *T*_max_ and *T*_min_ in Pakistan. In Iran, the SRAD was positively correlated with PREC and VAPR but did not correlated with longitude, latitude, *T*_max_, and *T*_min_ (Figure 4).

### 3.7 EigenGWAS for selective sweeps in Pakistan and Iranian landraces

**FIGURE 5:**
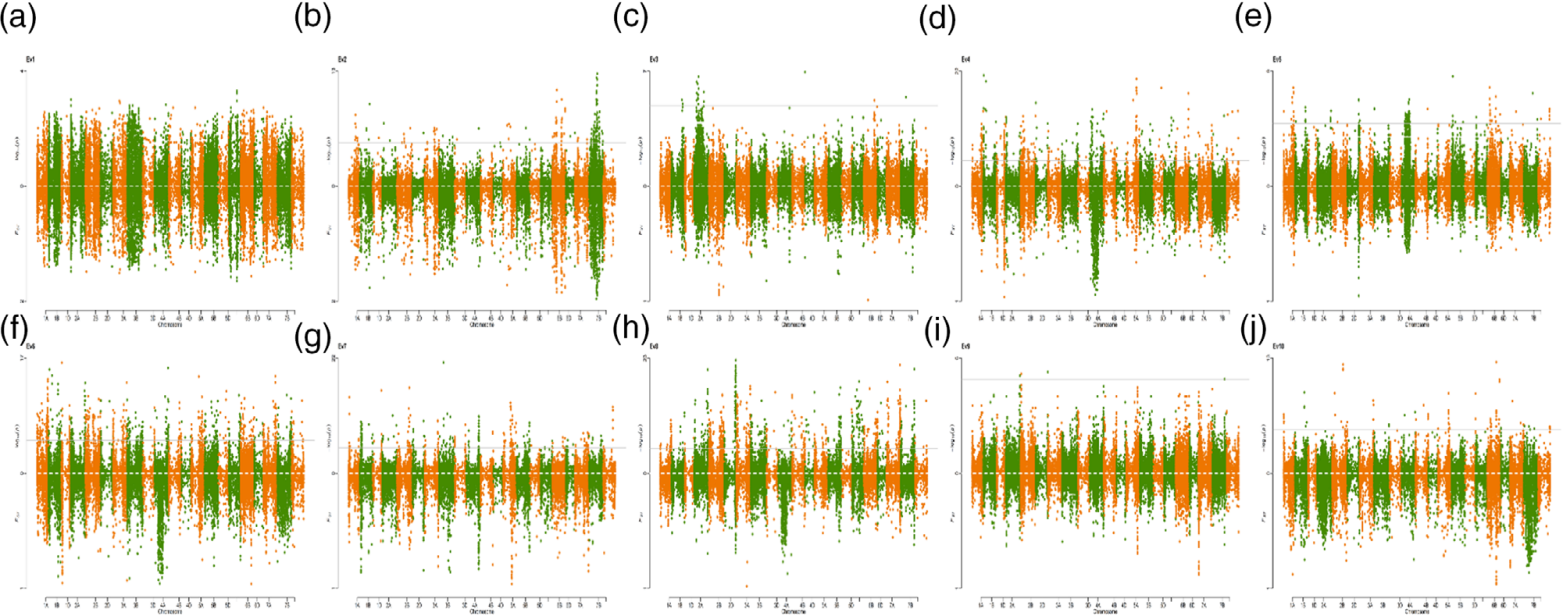
Miami plot from eigenvector decomposition genome‐wide association study (EigenGWAS) (upper for *P*_GC_ and lower for *Fst*) for eigenvector 1 (Ev1) to eigenvector 10 (Ev10) based on the 512 wheat landraces from Iran and Pakistan (a–j). *P*_GC_ is the *p*‐values corrected by λ_GC_ in EigenGWAS. Ev1–Ev10 are the first 10 eigenvectors, each of which was used as phenotype for the single‐marker association study based on nearly 43,868 single nucleotide polymorphism markers in EigenGWAS

EigenGWAS was conducted for the top 10 eigenvectors that explained 29.86% of the total genetic variation with each eigenvector explaining 0.83 to 12.36% of the genetic variation. The genomic inflation factor (λ_GC_) that is commonly used to adjust population stratification for GWAS (Devlin & Roeder, 1999) was calculated by EigenGWAS (Supplemental Table S7). EigenGWAS was conducted based upon positive or negative coordinates of the landraces from Iran and Pakistan on the corresponding eigenvector, and their selection differentiations were quantified by Fst. After correction by λ_GC_, the SNPs with −log_10_(*P*_GC_) that exceeded the threshold of 6.01 were declared as the loci under selection at the genome‐wide level. To facilitate the comparison, scanned results from −log_10_(*P*_GC_) and Fst were compared using a Miami plot (Figure 5a–j for each of the 10 eigenvectors). Generally, the peaks from −log_10_(*P*_GC_) and Fst mirrored each other, indicating reasonable grouping as defined by EigenGWAS. Overall, EigenGWAS identified selective sweeps on all 21 wheat chromosomes (Supplemental Table S7; Figure 5), while significant hits were found on 8 of the 10 eigenvectors except for Ev1 and Ev10. The highest number of hits were 760 SNPs over the three loci for Ev8. The distribution of the identified SNPs across different chromosomes varies considerably, with the highest number on chromosome 2B (207 SNPs) and the lowest on chromosome 4D (12 SNPs). The genomic region on chromosome 4A between 103 and 504 Mb consistently had several selective sweeps with more than 98 SNPs. All the selective sweeps identified by EigenGWAS were shared by the Fst scan. Selective sweeps were consistently detected in the three genomic regions of group 1 homeologous chromosomes flanking glutenin genes (Figure 5). Twenty‐five loci in selective sweeps were within close proximity (∼10 Mb) of functional genes including chromosome 1B (*TOE‐B1, TaFT3‐B1*, and *Elf3‐B1* at 6.06, 582.8, and 679.4 Mb, respectively, for flowering time [Zikhali et al., 2016, 2017]), chromosome 1D (*Mot3‐D1* and *Elf3‐D1* at 491.5 Mb for flowering time [Zikhali et al., 2017]), chromosome 3B (*Tamyb10‐B1* at 743.5 Mb for seed color [Himi et al., 2011]), chromosome 3D (*Tamyb10‐D1* at 556 Mb for seed color), chromosome 4A (*PRR73‐A1* at 120.1 Mb for flowering time and development [Zhang et al., 2016]), chromosome 4B (*PRR73‐B1* at 423 Mb [Zhang et al., 2016]), chromosome 4D (*Lr67* at 455.8 Mb for leaf rust resistance [Moore et al., 2015]), chromosome 5A (*Vrn‐A1* at 586.5 Mb for vernalization requirement), chromosome 5B (*Vrn‐B1* at 562.3 Mb), chromosome 6B (*SPL21‐6B* and *GW2‐6B* at 201 and 237.6 Mb, respectively, for grain size and weight [Yang et al., 2012]), chromosome 7A (*ALP‐7A* at 19.9 Mb for gluten strength [Chen et al., 2016] and *SPL20‐7A* at 692.4 Mb for grain size [Zhang et al., 2017]), chromosome 7B (*Pinb2* at 703.9 Mb for grain hardness [Geng et al., 2012]), and chromosome 7D (*TaGS3‐D1* and *SPL20‐7D* at 5.82 and 515.8 Mb, respectively, for grain size).

### 3.8 GWAS for local adaptability based on ACVs

**FIGURE 6:**
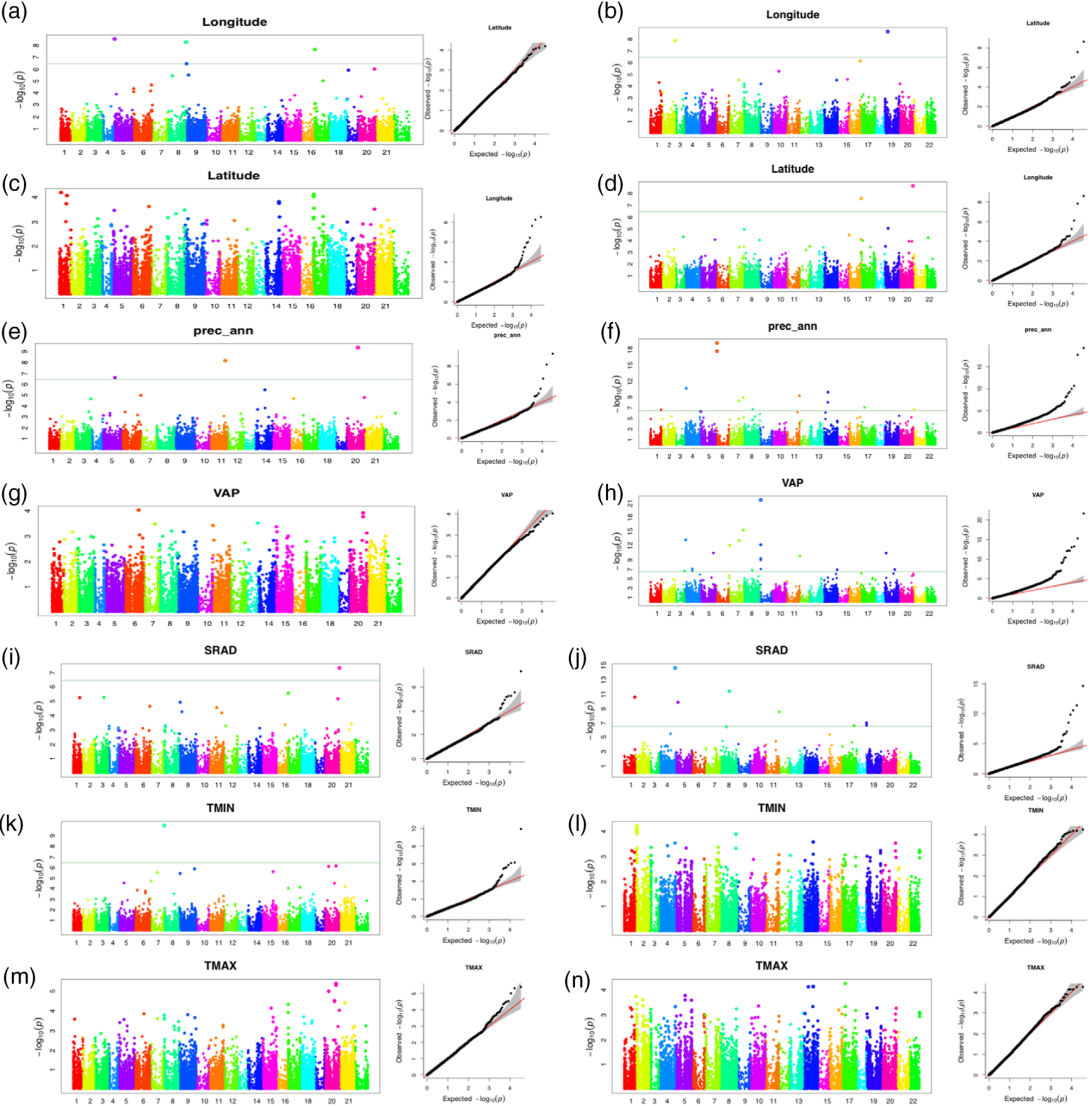
Manhattan and quantile–quantile plot showing significant marker‐trait associations in landraces from Pakistan (left panel) and Iran (right panel), for the agro‐climatic variables (a and b) longitude, (c and d) latitude, (e and f) annual precipitation (prec_ann), (g and h) vapors (VAP), (i and j) solar radiations (SRAD), (j and k) minimum temperature (*T*_min_), and (m and n) maximum temperature (*T*_max_)

The GWAS for ACVs identified 90 loci in Iranian landraces and 58 loci in Pakistani landraces (Figure 6a–k for each variable). The Manhattan and Q–Q‐plots are shown for each trait like longitude (Figure 6a for Pakistan and Figure 6b for Iran), and vice versa, for each trait. The maximum number of loci associated with ACVs were identified on chromosome 7B (16) and minimum on chromosome 4D (2). The maximum number of loci were identified for VAPR (45) and the minimum number of loci were identified for *T*_max_ (8). Among these, 17 loci were associated with multiple variables (Supplemental Table S7). For example, four loci on chromosome 6B (13.7 Mb), 7B (193.4 and 717. 2 Mb), and 7D (159.7 Mb) were associated with both *T*_min_ and *T*_max_. Similarly, a locus on chromosome 6B (75.9 Mb) was associated with latitude and SRAD in the Pakistani subpopulation. Based on the colocalization of loci within 10 Mb proximity, the common loci in both subpopulations include those for longitude on chromosome 3D, annual precipitation on chromosome 4B (650 Mb), SRAD on chromosome 3D (3.3 Mb), and VAPR on chromosome 3B (∼41 Mb). Similarly, based on colocalization, six loci were within proximity of selective sweeps, which included those for VAPR on chromosome 1D, 5D in the Iranian subpopulation and 5D in the Pakistani subpopulation, and precipitation on 2A and 6B in the Iranian subpopulation.

Some functional genes were also identified within close proximity of loci associated with ACVs. These include *Elf3‐B1* (Wang et al., 2016), a flowering time‐related gene, in close proximity to 1B locus associated with longitude at the 678.2 Mb position. *Vrn‐D1* was also found in proximity of a locus associated with longitude among Iranian landraces. Similarly, a locus associated with longitude among Pakistani landraces was within close proximity to *Ppd‐D1* locus, a photoperiod responsive gene, on chromosome 2D at position 2.7 Mb. The same locus was also associated with PREC among Iranian landraces. A drought‐related gene, *TaCwi‐4A*, was identified in proximity of the locus associated with VAPR on chromosome 4A at position 647.8 Mb among Iranian landraces (Jiang et al., 2015; Webster et al., 2012).

## 4 DISCUSSION

### 4.1 Genetic diversity and population structure

The present study investigated a panel of wheat landraces from two important geographical regions for wheat domestication, Pakistan and Iran. That the B genome had the highest number of SNPs in the panel of landraces was consistent with some previous studies (Alipour et al., 2017; Chao et al., 2007). The higher percentage of Ts SNPs (64.29%) than Tv SNPs (35.71%) in the current study is comparable with 64.5% of transition SNPs and 35.5% of transversion SNPs reported by Winfield et al. (2016). The significantly higher number of Ts SNPs than Tv SNPs was due to shifting of methylcytosine to uracil and then mutating to thymine. The hexaploid wheat genome is highly methylated because of its polyploidy, which may explain the higher number of transition SNPs in wheat. The higher Ts/Tv SNP ratio improves the accuracy for SNP prediction with a greater level of confidence.

Genetic variability in the wheat landrace panel revealed by PIC and genetic diversity reflected genetic diversity in a genomic level or polymorphism in DNA fragments of a population and may facilitate the evolutionary process by creating the selection pressure on the alternate forms of genes. The mean PIC value (0.162) estimated in wheat landraces in this study was consistent with Chao et al. (2007) and Ren et al. (2013), but lower than that (0.30) estimated by Liu et al. (2017). In the present study, the Pakistani subpopulation had a higher gene diversity than the Iranian subpopulation. The biallelic nature of most of the SNPs may be one of the reasons for overall low genetic diversity compared with that estimated by simple sequence repeats markers (Chao et al., 2007). The differences in diversity between Pakistani and Iranian landraces indicated that the Pakistani subpopulation was relatively more diverse. The genetic variation present among and within groups can be used for wheat improvements in breeding programs.

It is important to accurately analyze and define the relationship between the molecular and functional diversities in natural populations based on the structural analyses of a particular population (Buckler & Thornsberry, 2002). The population structure analyses classified the landrace panel into two subpopulations with the highest Δ*K* value of 2. The structure plot, cluster analysis, and PCA confirmed the classification of the panel into two lineages with some international varieties from the United States, Mexico, and Italy in admixture with Pakistani germplasm. The cluster analyses using the SNPs from each genome separately gave the same results and clearly separated the panel into Pakistan and Iran subpopulations, suggesting that the two landrace subpopulations have different genetic makeups. The genetic diversity found between the two subpopulations could be the results of different selection pressures imposed by native people and cultivating habits (Alipour et al., 2017). The higher Fst value (*p* < .0001) between the Pakistani and Iranian subpopulations suggests higher genetic differentiations between the two subpopulations, and most of the genetic differences between these landraces occur on the B genome.

### 4.2 LD in landraces

The LD is the basis for association mapping. Linkage disequilibrium has been extensively studied in bread wheat at both the full genome and individual chromosomal levels (Chao et al., 2009). In this study, we examined LD and LD decay at both levels of the wheat landrace panel. About 26.97% of SNP pairs were in significant LD (*p* < .001), which was similar to the LD (22%) found in maize (*Zea mays* L.) inbred lines (Dinesh et al., 2016). The current study also revealed that the B genome (especially chromosomes 2B and 3B) had the highest LD, and the D genome (specifically 4D) had the lowest LD. The number of SNP pairs in the LD in the D genome of the Iranian genotypes was much higher than that for Pakistani genotypes. Also, the D genome had a longer genetic distance of significant LD than A and B genomes for both the Pakistani (17.67 Mb) and Iranian (18.87 Mb) subpopulations. The results of the LD and LD decay in Pakistani and Iranian wheat landrace subpopulations may be used as a foundation to determine the proper marker density for quantitative trait loci (QTL) mapping and markers linked to QTLs for marker‐assisted wheat breeding. Comparison of the LD decay distances between the Iranian and Pakistani wheat landraces revealed that Pakistani landraces had the higher *r* ^2^ values for all the three genomes than those for Iranian landraces. The variation observed at the chromosomal LD decay distances was positively correlated with the variation in recombination rates in previous studies (Dooner & He, 2008).

### 4.3 Selective sweeps and GWAS for local adaptability

The identification of selected regions is a basic step in understanding adaptability and selection history (Helyar et al., 2011; Narum & Hess, 2011). The genes and SNPs located in these regions should have been under selection and are valuable for breeding and for germplasm selection. In this study, selective regions between landraces in Pakistan and Iran were determined among genomes using EigenGWAS. Chen et al. (2016) proposed a single‐marker regression approach based on PCA (or eigenanalysis). The regression coefficient of EigenGWAS was approximately equal to Fst. In recent studies, EigenGWAS has been successfully deployed to identify selection signals in species, such as humans (Parolo et al., 2017) and wild birds (Bosse et al., 2017; Kim et al. 2017). They identified the genes under selection or adaptation and illustrated how genetic signatures of selection can be translated into variation in fitness and phenotypes. In our study, a total of 122 selected regions were found in both landraces sets, out of which 26 were colocalized with important developmental genes. Cavanagh et al. (2013) identified selection regions using the wheat 9K SNP arrays in 2,994 hexaploid wheat accessions and found that most selected regions were associated with yield potential, vernalization, plant height, and biotic and abiotic stress tolerance. Zhou et al. (2018 investigated 717 Chinese wheat landraces with 27,933 diversity arrays technology and 312,831 SNP markers and found 148 selected regions associated with yield and disease tolerance. The selected regions in our study were associated with important agronomic traits, including genes for yield‐related traits (*TaGS3‐D1*, *TaGW2‐6B*, *SPL20‐7D*, and *SPL20‐7A*), end‐use quality (*ALP‐7A, Pinb2*, and *Tamyb10*), disease resistance (*Lr67*), and vernalization (*Vrn‐A1, Vrn‐D4*, and *Vrn‐B1*). The allelic variation for most of the genes, such as *SPL20‐7A, 7D, ALP‐7A, Tamyb10*, and *TaGS3‐D1*, is unknown in most of the geographic regions of the world. Zhao et al. (2019) surveyed allelic variation of 47 functional genes in a global wheat collection of 1,152 accessions from 21 countries. It is the only major germplasm survey study available to compare the frequency of functional genes; however, the germplasm collection was mainly composed of improved cultivars. From their results, it was quite clear that frequency of favorable alleles for yield‐related genes like *TaGW2* and *TaGS‐D1* was quite higher in improved cultivars compared with landraces. The GWAS hits near glutenin and *Tamyb10* are plausible because the end‐use preference in Iran and Pakistan has remained different. In Pakistan, wheat is used most often to prepare flatbread or chapatti (Rasheed et al., 2015), which is different from wheat products in Iran such as lavash, barbari, and sangak. The products from both countries require different glutenin and grain texture quality, which are primarily controlled by *Glu‐1, Glu‐3*, and *Pin* loci. Some evidence from the literature supports our results; for example, *Lr67* is a durable rust resistance gene evolved after wheat polyploidization (Moore et al., 2015), and its resistance allele frequency is predominant in landraces from Pakistan and India (Riaz et al., 2016). Similarly, Kippes et al. (2015) identified a *Vrn‐D4* gene, which was important for the development of spring growth habit in the ancient wheats (*T. aestivum* L. ssp. *sphaerococcum*) from South Asia. Although the presence of *Vrn‐D4* in *T. aestivum* landraces is not yet surveyed, it is likely that the genomic region spanning *Vrn‐D4* on chromosome 5DS is under differential selection compared with Iranian landraces.

Modern breeding has effectively improved wheat adaptation to human preferences and various environments. Recently, many studies have been done to dissect the meaningful association between genotypic polymorphisms and environmental variables in natural populations by using different statistical tools (Ferrero‐Serrano & Assmann, 2019; Günther et al., 2016; Russell et al., 2016). The use of genome–environment associations will help us understand crop adaptation to certain environments. However, little research has been reported on this topic because of the lack of reliable germplasm passport data, proper geographical coverage in germplasm collections, and undisputed genetic grouping (Contreras‐Moreira et al., 2019). The data for ACVs in the current study was collected from extremely fine grids of weather data to uncover polygenic adaptation and domestication in wheat. The environmental GWAS (EnvGWAS) in this study used precise geographical, climatic, and soil data for each landrace collection site (referred to as GIS data for simplicity) and genomic data for those wheat landraces (Navarro et al., 2017), which provides important information to further decipher the genomic loci and regions affecting wheat adaptation. As GIS data are highly associated with crop selection and adaptation, they are also associated with population structure (Li et al., 2019).

In crop improvement, genome–environment associations may be integrated with genome–phenotype associations to select for climate resilience traits (Lasky et al., 2015). Previously, genetic loci were identified for adaptive traits in soybean [*Glycine max* (L.) Merr.] landraces with 27 SNPs associated with three ACVs, i.e., temperature, precipitation, and day length (Li et al., 2020). Li et al. (2019) uncovered polygenic adaptation traits in 1,143 maize accessions using EnvGWAS and identified a large number of loci associated with ACVs. In our study, many loci that associated with flowering time‐related genes (*Vrn‐1, Ppd1*, and *Elf3‐B*1) were expected. Flowering time is a critical adaptive trait that affects reproductive success in a local environment and is typically studied in relation to environmental factors such as changes in temperature and daylengths (Amasino & Michaels, 2010). In our study, flowering time‐related genes were detected within LD blocks surrounding five loci. The early flowering gene *TaElf3‐B1* was within the LD block of a locus associated with longitude among Iranian landraces on chromosome 1B; *Ppd‐D1* was close to a locus on chromosome 2D associated with longitude in Pakistani landraces and precipitation in Iranian landraces. Previously, Jones et al. (2013) reported that diversity at *Ppd‐D1* locus in *Aegilops tauschii* explained the species distribution along geographic coordinates, and that allelic variation is associated with adaptability pattern. Another developmental‐related gene, *Rht‐8*, is linked with *Ppd‐D1*; the former is among the so‐called Green Revolution genes inducing short stature in wheat germplasm. *Vrn‐A1* was close to a locus associated with VAPR on chromosome 5A in Pakistani landraces, and *Vrn‐D1* was within LD of a locus associated with longitude in Iranian landraces. Previously, maize landraces were used to identify the genomic region controlling the adaptability to longitude and latitude, and it was further revealed that about 61.1% of SNPs for altitude were associated with flowering time (Navarro et al., 2017). Similarly, SNPs associated with longitude on chromosome 1B were in close proximity of the *Elf3‐B1* gene, which is an important early flowering inducing gene. Some important mutations in *Vrn‐A1* and *Vrn‐B3* genes have been identified in landraces from Pakistan and Iran. Sehgal et al. (2015) identified the *Vrn‐A1f* allele, which is a deletion of 5,997 bp within the *Vrn‐A1* gene, in landraces from Iran, Iraq, and Afghanistan, thus pointing to a Middle Eastern and/or near eastern origin of this allele. Derakhshan et al. (2013) reported an 890‐bp insertion in the *vrn‐B3* gene from Iranian landraces, and another allele, *Vrn‐B3b*, was identified in landraces from Pakistan, Iran, Uzbekistan, Turkmenistan, and Afghanistan, indicating a wide distribution of this allele. Similarly, SNPs in close proximity of the *Vrn‐1* loci have been associated with important ACVs like longitude, latitude, and VAPR in both Iranian and Pakistani landraces.

Temperature is another well‐known climatic factor affecting plant growth and influencing plant distribution and productivity (Lobréaux & Melodelima, 2015). Because temperature is an influential factor for crop adaptation, it has been associated with some genomic regions. Lobréaux and Melodelim (2015) found that 25 genomic regions on four different chromosomes were associated with *T*_min_ in *Arabidopsis thaliana*. It is likely that two loci in Iranian landraces (on chromosomes 5B and 6B) and six loci in Pakistani landraces (on chromosomes 5B, 6D, 7B, and 7D) that were associated with *T*_max_ could be important for heat tolerance because they explained the adaptability of landraces in the geographic regions with the highest temperatures. Because the temperature gradients are different between the two countries, we could not find any common loci associated with temperature attributes between the two subpopulations.

The absence of common loci associated with ACV is expected and reported in literature. He et al. (2019) correlated the genomic regions associated with bioclimatic variables in different wheat populations and identified 43,670 genomic regions covering about 988 Mbs of the wheat genome. Cross‐population comparisons indicated that most genomic regions in selective sweeps do not overlap, and only three genomic regions were shared among all nine populations used in the comparison. Because both populations in our study were not an admixture, it was difficult to find common genetic basis of wheat adaptability to high temperature in both populations. The reasons for the significant variation among populations could be that (a) selection pressures are likely to vary temporally and spatially, and strongly influenced by environmental conditions, such as climatic regime, biotic and abiotic stress, altitude, annual sunlight, temperature, and precipitation; (b) genotypes underwent different selection pressures to adapt to local agricultural practices; and (c) selection may be acting on multiple functionally equivalent mutations in different populations (Cavanagh et al., 2013; Ralph & Coop, 2010).

Several loci with significant effects on adaptation may not be detected due to the strict quality‐control procedure of filtering minor alleles, which is a common limitation of GWAS. Such loci can be detected in the biparental populations by QTL mapping in populations derived from the cross of landraces and cultivars. However, Boyle et al. (2017) indicated that species generally adapt by small allele frequency shifts of many causal genes across the genome; hence, the GWAS could be the best approach to identify those variants with moderate‐to‐minor effects. The reported adaptation loci could have applications in breeding if the favored allele frequency is lower in modern wheat cultivars. Because these loci could explain environmental adaptability, high breeding value landraces can be identified by having a high frequency of favored alleles. These results will help breeders better understand the implication of the genomic footprint on crop adaptation and plant responses to climate changes and better explore exotic germplasm sources in a more targeted manner. We are currently extending this and genomic prediction‐based approaches to the whole germplasm bank using genomic and GIS data to identify genomic regions and accessions of highest breeding relevance.

## AUTHOR CONTRIBUTIONS

AR, HL, and AG designed the research; GB provided reagents and facility for genotyping; AG, FM, and RA conducted genotyping; UH, AR, HL, and JL performed data analyses; KI, AG, SUS, HA, MRB, VM, and SAP contributed germplasm; AR, UH, and HA wrote the manuscript; all authors edited and approved the final version of the manuscript. Uzma Hanif: Investigation; Writing‐original draft. Hadi Alipour: Data curation; Formal analysis; Resources. Alvina Gul: Formal analysis; Funding acquisition; Software; Writing‐original draft. Li Jing: Formal analysis; Methodology; Software. Reza Darvishzadeh: Writing‐review & editing. Rabia Amir: Investigation; Writing‐review & editing. Faiza Munir: Formal analysis; Writing‐review & editing. Sadar Uddin Siddiqui: Resources. Muhammad Kashif Ilyas: Resources. Abdul Ghafoor: Resources. Paul St. Amand: Resources; Supervision; Writing‐review & editing. Guihua Bai: Funding acquisition; Supervision; Writing‐review & editing. Kai Sonder: Data curation; Formal analysis; Software. Zhonghu He: Funding acquisition; Project administration; Writing‐review & editing. Awais Rasheed: Formal analysis; Project administration; Writing‐original draft; Writing‐review & editing. Huihui Li: Formal analysis; Supervision; Writing‐original draft; Writing‐review & editing.

## CONFLICT OF INTEREST

All authors declare no conflict of interest.

## Supporting information

Table S1: The list of Pakistani wheat landraces collected between 1931 and 1968 and their ecological zones
Table S2: The list of Iranian wheat landraces collected between 1931 and 1968 and their ecological zones
Table S3. Summary of single nucleotide substitutions identified in three homoeologous genomes in reference to the IWGSC wheat reference genome version 1.0 in landraces from Iran and Pakistan
Table S4. Summary of single nucleotide substitutions identified in each chromosome in reference to the IWGSC wheat reference genome version 1.0 in landraces from Iran and Pakistan
Table S5. Co‐efficient of correlation among agro‐climatic traits (monthly average) from Pakistan (lower diagonal) and Iran (upper diagonal)
Table S6. EigenGWAS analysis indicating the loci under positive selection in landraces from Pakistan and Iran.
Table S7. Marker‐trait associations for agro‐climaric variables based on the analysis of landraces from Iran, Pakistan and combined from both countries. This include the SNP name, chromosome position, p‐value, minor allele frequency (maf), allelic effect of minor allele and co‐localization information with functional gene or selection loci
Table S8: Genotypic data for Iranian and Pakistani accessions in hapmap format
Table S9: Phenotypic data of all wheat accessions used in the study

## Data Availability

The genotypic and phenotypic data is presented as Supplemental Tables S8 and S9, respectively.

## References

[tpg220096-bib-0001] Alipour, H. , Bihamta, M. R. , Mohammadi, V. , Peyghambari, S. A. , Bai, G. , & Zhang, G. (2017). Genotyping‐by‐sequencing (GBS) revealed molecular genetic diversity of Iranian wheat landraces and cultivars. Frontiers in Plant Science, 8, 1293. 10.3389/fpls.2017.01293 28912785 PMC5583605

[tpg220096-bib-0002] Amasino, R. M. , & Michaels, S. D. (2010). The timing of flowering. Plant Physiology, 154, 516. 10.1104/pp.110.161653 20921176 PMC2948982

[tpg220096-bib-0003] Bosse, M. , Spurgin, L. G. , Laine, V. N. , Cole, E. F. , Firth, J. A. , Gienapp, P. , Gosler, A. G. , McMahon, K. , Poissant, I. , & Groenen, M. A. (2017). Recent natural selection causes adaptive evolution of an avian polygenic trait. Science, 358, 365–368. 10.1016/j.cell.2017.05.038 29051380

[tpg220096-bib-0004] Boyle, E. A. , Li, Y. L. , & Pritchard, J. K. (2017). An expanded view of complex traits: From polygenic to omnigenic. Cell, 169, 1177–1186. 10.1016/j.cell.2017.05.038 28622505 PMC5536862

[tpg220096-bib-0005] Bradbury, P. J. , Zhang, Z. , Kroon, D. E. , Casstevens, T. M. , Ramdoss, Y. , & Buckler, E. S. (2007) TASSEL: Software for association mapping of complex traits in diverse samples. Bioinformatics, 23, 2633–2635. 10.1093/bioinformatics/btm308 17586829

[tpg220096-bib-0006] Buckler, E. S. , & Thornsberry, J. M. (2002). Plant molecular diversity and applications to genomics. Current Opinion in Plant Biology, 5, 107–111. 10.1016/S1369-5266(02)00238-8 11856604

[tpg220096-bib-0007] Cavanagh, C. R. , Chao, S. M. , Wang, S. C. , Huang, B. E. , Stephen, S. , Kiani, S. , Forrest, K. , Saintenac, C. , Brown‐Guedira, G. L. , Akhunova, A. , See, D. , Bai, G. H. , Pumphrey, M. , Tomar, L. , Wong, D. B. , Kong, S. , Reynolds, M. , da Silva, M. L. , Bockelman, H. , … Akhunov, E. (2013). Genome‐wide comparative diversity uncovers multiple targets of selection for improvement in hexaploid wheat landraces and cultivars. Proceedings of the National Academy of Sciences of the United States of America, 110, 8057–8062. 10.1073/pnas.1217133110 23630259 PMC3657823

[tpg220096-bib-0008] Chang, C. C. , Chow, C. C. , Tellier, L. C. , Vattikuti, S. , Purcell, S. M. , & Lee, J. J. (2015). Second‐generation PLINK: Rising to the challenge of larger and richer datasets. Gigascience, 4, 7. 10.1186/s13742-018-0047-8 25722852 PMC4342193

[tpg220096-bib-0009] Chao, S. M. , Zhang, W. J. , Akhunov, E. , Sherman, J. , Ma, Y. Q. , Luo, M. C. , & Dubcovsky, J. (2009). Analysis of gene‐derived SNP marker polymorphism in US wheat (*Triticum aestivum* L.) cultivars. Molecular Breeding, 23, 23–33. 10.1007/s11032-008-9210-6

[tpg220096-bib-0010] Chao, S. M. , Zhang, W. J. , Dubcovsky, J. , & Sorrells, M. (2007). Evaluation of genetic diversity and genome‐wide linkage disequilibrium among US wheat (*Triticum aestivum* L.) germplasm representing different market classes. Crop Science, 47, 1018–1030. 10.2135/cropsci2006.06.0434

[tpg220096-bib-0011] Chen, X. Y. , Cao, X. Y. , Zhang, Y. J. , Islam, S. , Zhang, J. J. , Yang, R. C. , Liu, J. J. , Li, G. Y. , Appels, R. , Keeble‐Gagnere, G. , Ji, W. Q. , He, Z. H. , & Ma, W. J. (2016). Genetic characterization of cysteine‐rich type‐b avenin‐like protein coding genes in common wheat. Scientific Reports, 6, 30692. 10.1038/srep30692 27503660 PMC4977551

[tpg220096-bib-0012] Contreras‐Moreira, B. , Serrano‐Notivoli, R. , Mohammed, N. E. , Cantalapiedra, C. P. , Beguería, S. , Casas, A. M. , & Igartua, E. (2019). Genetic association with high‐resolution climate data reveals selection footprints in the genomes of barley landraces across the Iberian Peninsula. Molecular Ecology, 28, 1994–2012. 10.1111/mec.15009 30614595 PMC6563438

[tpg220096-bib-0013] Derakhshan, B. , Mohammadi, S. A. , Moghaddam, M. , & Jalal Kamali, M. R. (2013). Molecular characterization of vernalization genes in Iranian wheat landraces. Crop Breeding Journal, 3, 11–14.

[tpg220096-bib-0014] Devlin, B. , & Roeder, K. (1999). Genomic control for association studies. Biometrics, 55, 997–1004. 10.1111/j.0006-341x.1999.00997.x 11315092

[tpg220096-bib-0015] Dinesh, A. , Patil, A. , Zaidi, P. H. , Kuchanur, P. H. , Vinayan, H. T. , & Seetharam, K. (2016). Genetic diversity, linkage disequilibrium and population structure among CIMMYT maize inbred lines, selected for heat tolerance study. Mydica, 61, 216.

[tpg220096-bib-0016] Dooner, H. K. , & He, L. (2008). Maize genome structure variation: Interplay between retrotransposon polymorphisms and genic recombination. The Plant Cell, 20, 249. 10.1105/tpc.107.057596 18296625 PMC2276454

[tpg220096-bib-0017] Dvorak, J. , Luo, M. C. , & Akhunov, E. D. (2011). NI Vavilov's theory of centres of diversity in the light of current understanding of wheat diversity, domestication and evolution. Czech Journal of Genetics and Plant Breeding, 47, S20–S27. 10.17221/3249-CJGPB

[tpg220096-bib-0018] Dvorak, J. , Luo, M. C. , Yang, Z. L. , & Zhang, H. B. (1998) The structure of the Aegilops tauschii genepool and the evolution of hexaploid wheat. Theoretical and Applied Genetics, 97, 657–670. 10.1007/s001220050942

[tpg220096-bib-0019] Dwivedi, S. L. , Ceccarelli, S. , Blair, M. W. , Upadhyaya, H. D. , Are, A. K. , & Ortiz, R. (2016). Landrace germplasm for improving yield and abiotic stress adaptation. Trends in Plant Science, 21, 31–42. 10.1016/j.tplants.2015.10.012 26559599

[tpg220096-bib-0020] Environmental System Research Institute . (2018). *ArcMap* (Version 10.6) [Software]. ESRI. https://desktop.arcgis.com/en/arcmap/10.6/extensions/aviation‐charting/what‐is‐the‐arcgis‐for‐aviation.htm

[tpg220096-bib-0021] Evanno, G. , Regnaut, S. , & Goudet, J. (2005). Detecting the number of clusters of individuals using the software STRUCTURE: A simulation study. Molecular Ecology, 14, 2611–2620. 10.1111/j.1365-294X.2005.02553.x 15969739

[tpg220096-bib-0022] Ferrero‐Serrano, Á. , & Assmann, S. M. (2019). Phenotypic and genome‐wide association with the local environment of *Arabidopsis* . Nature Ecology & Evolution, 3, 274–285. 30643246 10.1038/s41559-018-0754-5

[tpg220096-bib-0023] Fick, S. E. , & Hijmans, R. J. (2017). WorldClim 2: New 1‐km spatial resolution climate surfaces for global land areas. International Journal of Climatology, 37, 4302–4315. 10.1002/joc.5086

[tpg220096-bib-0024] Fuller, D. Q. (2006). Agricultural origins and frontiers in South Asia: A working synthesis. Journal of World Prehistory, 20, 1–86. 10.1007/s10963-006-9006-8

[tpg220096-bib-0025] Geng, H. W. , Beecher, B. S. , He, Z. H. , & Morris, C. F. (2012). Physical mapping of Puroindoline b‐2 genes in wheat using ‘Chinese Spring’ chromosome group 7 deletion lines. Crop Science, 52, 2674–2678. 10.2135/cropsci2012.04.0241

[tpg220096-bib-0026] Günther, T. , Lampei, C. , Barilar, I. , & Schmid, K. J. (2016). Genomic and phenotypic differentiation of *Arabidopsis thaliana* along altitudinal gradients in the North Italian Alps. Molecular Ecology, 25, 3574–3592. 10.1111/mec.13705 27220345

[tpg220096-bib-0027] He, F. , Pasam, R. , Shi, F. , Kant, S. , Keeble‐Gagnere, G. , Kay, P. , Forrest, K. , Fritz, A. , Hucl, P. , Wiebe, K. , Knox, R. , Cuthbert, R. , Pozniak, C. , Akhunova, A. , Morrell, P. L. , Davies, J. P. , Webb, S. R. , Spangenberg, G. , Hayes, B. , … Akhunov, E. (2019). Exome sequencing highlights the role of wild‐relative introgression in shaping the adaptive landscape of the wheat genome. Nature Genetics, 51, 896–904. 10.1038/s41588-019-0382-2 31043759

[tpg220096-bib-0028] Hengl, T. , Mendes de Jesus, J. , Heuvelink, G. B. M. , Gonzalez, M. R. , Kilibarda, M. , Blagotić, A. , Shangguan, W. , Wright, M. N. , Geng, X. , Bauer‐Marschallinger, B. , Guevara, M. A. , Vargas, R. , MacMillan, R. A. , Batjes, N. H. , Leenaars, J. G. B. , Ribeiro, E. , Wheeler, I. , Mantel, S. , & Kempen, B. (2017). SoilGrids250m: Global gridded soil information based on machine learning. PLOS ONE, 12, e0169748. 10.1371/journal.pone.0169748 28207752 PMC5313206

[tpg220096-bib-0029] Helyar, S. J. , Hemmer‐Hansen, J. , Bekkevold, D. , Taylor, M. I. , Ogden, R. , Limborg, M. T. , Cariani, A. , Maes, G. E. , Diopere, E. , Carvalho, G. R. , & Nielsen, E. E. (2011). Application of SNPs for population genetics of nonmodel organisms: New opportunities and challenges. Molecular Ecology Resources, 11, 123–136. 10.1111/j.1755-0998.2010.02943.x 21429169

[tpg220096-bib-0030] Himi, E. , Maekawa, M. , Miura, H. , & Noda, K. (2011). Development of PCR markers for Tamyb10 related to R‐1, red grain color gene in wheat. Theoretical and Applied Genetics, 122, 1561–1576. 10.1007/s00122-011-1555-2 21359957

[tpg220096-bib-0031] International Wheat Genome Sequencing Consortium. (2018). Shifting the limits in wheat research and breeding using a fully annotated reference genome. Science, 361, eaar7191. 10.1126/science.aar7191 30115783

[tpg220096-bib-0032] Jiang, Y. , Jiang, Q. , Hao, C. , Hou, J. , Wang, L. , Zhang, H. , Zhang, S. , Chen, X. , & Zhang, X. (2015). A yield‐associated gene TaCWI, in wheat: Its function, selection and evolution in global breeding revealed by haplotype analysis. Theoretical and Applied Genetics, 128, 131–143. 10.1007/s00122-014-2417-5 25367379

[tpg220096-bib-0033] Jones, H. , Gosman, N. , Horsnell, R. , Rose, G. A. , Everest, L. A. , Bentley, A. R. , Tha, S. , Uauy, C. , Kowalski, A. , Novoselovic, D. , Simek, R. , Kobiljski, B. , Kondic‐Spika, A. , Brbaklic, L. , Mitrofanova, O. , Chesnokov, Y. , Bonnett, D. , & Greenland, A. (2013). Strategy for exploiting exotic germplasm using genetic, morphological, and environmental diversity: The *Aegilops tauschii* Coss. example. Theoretical and Applied Genetics, 126, 1793–1808. 10.1007/s00122-013-2093-x 23558983

[tpg220096-bib-0034] Jordan, K. W. , Wang, S. , Lun, Y. , Gardiner, L.‐ J. , MacLachlan, R. , Hucl, P. , Wiebe, K. , Wong, D. , Forrest, K. L. , International Wheat Genome Sequencing Consortium, Sharpe, A. G. , Sidebottom, C. H. , Hall, N. , Toomajian, C. , Close, T. , Dubcovsky, J. , Akhunova, A. , Talbert, L. , Bansal, U. K. , … Akhunov, E. (2015). A haplotype map of allohexaploid wheat reveals distinct patterns of selection on homoeologous genomes. Genome Biology, 16, 48. 10.1186/s13059-015-0606-4 25886949 PMC4389885

[tpg220096-bib-0035] Kihara, H. , Yamashita, H. , & Tanaka, M. (1965). Morphological, physiological, genetical, and cytological studies in Aegilops and Triticum collected in Pakistan, Afghanistan, Iran. In K. Yamashita (Ed.), *Results of Cultivated plants and their relatives. The Kyoto University scientific expedition to the Korakoram and Hindukush in 1955* (Vol. 1). Kyoto University.

[tpg220096-bib-0036] Kippes, N. , Debernardi, J. M. , Vasquez‐Gross, H. A. , Akpinar, B. A. , Budak, H. , Kato, K. , Chao, S. , Akhunov, E. , & Dubcovsky, J. (2015). Identification of the *VERNALIZATION 4* gene reveals the origin of spring growth habit in ancient wheats from South Asia. Proceedings of the National Academy of Sciences, 112, E5401. 10.1073/pnas.1514883112 PMC4593092 26324889

[tpg220096-bib-0037] Lasky, J. R. , Upadhyaya, H. D. , Ramu, P. , Deshpande, S. , Hash, C. T. , Bonnette, J. , Juenger, T. E. , Hyma, K. , Acharya, C. , Mitchell, S. E. , Buckler, E. S. , Brenton, Z. , Kresovich, S. , & Morris, G. P. (2015). Genome‐environment associations in sorghum landraces predict adaptive traits. Science Advances, 1, e1400218. 26601206 10.1126/sciadv.1400218 PMC4646766

[tpg220096-bib-0038] Li, J. , Chen, G.‐B. , Rasheed, A. , Li, D. , Sonder, K. , Zavala Espinosa, C. , Wang, J. , Costich, D. E. , Schnable, P. S. , Hearne, S. J. , & Li, H. (2019). Identifying loci with breeding potential across temperate and tropical adaptation via EigenGWAS and EnvGWAS. Molecular Ecology, 28, 3544–3560. 10.1111/mec.15169 31287919 PMC6851670

[tpg220096-bib-0039] Li, Y. ‐H., Li, D. , Jiao, Y.‐Q. , Schnable, J. C. , Li, Y.‐F. , Li, H.‐H. , Chen, H.‐Z. , Hong, H.‐I. , Zhang, T. , Liu, B. , Liu, Z.‐X. , You, Q.‐B. , Tian, Y. , Guo, Y. , Guan, R.‐X. , Zhang, L.‐J ., Chang, R.‐Z. , Zhang, Z. , Reif, J. , … Qiu, L.‐J. (2020). Identification of loci controlling adaptation in Chinese soya bean landraces via a combination of conventional and bioclimatic GWAS. Plant Biotechnology Journal, 18, 389–401. 10.1111/pbi.13206 31278885 PMC6953199

[tpg220096-bib-0040] Liu, X. , Huang, M. , Fan, B. , Buckler, E. S. , & Zhang, Z. (2016). Iterative usage of fixed and random effect models for powerful and efficient genome‐wide association studies. PLOS Genetics, 12, e1005767. https://www.journal.pgen.1005767 26828793 10.1371/journal.pgen.1005767 PMC4734661

[tpg220096-bib-0041] Liu, Y. , Lin, Y. , Gao, S. , Li, Z. , Ma, J. , Deng, M. , Chen, G. , Wei, Y. , & Zheng, Y. (2017). A genome‐wide association study of 23 agronomic traits in Chinese wheat landraces. The Plant Journal, 91, 861–873. 10.1111/tpj.13614 28628238

[tpg220096-bib-0042] Lobréaux, S. , & Melodelima, C. (2015). Detection of genomic loci associated with environmental variables using generalized linear mixed models. Genomics, 105, 69–75. 10.1016/j.ygeno.2014.12.001 25499197

[tpg220096-bib-0043] Lopes, M. S. , Baenziger, P. S. , El‐Basyoni, I. , Vikram, P. , Singh, S. , Royo, C. , Ozbek, K. , Aktas, H. , Ozer, E. , Ozdemir, F. , Manickavelu, A. , & Ban, T. (2015). Exploiting genetic diversity from landraces in wheat breeding for adaptation to climate change. Journal of Experimental Botany, 66, 3477–3486. 10.1093/jxb/erv122 25821073

[tpg220096-bib-0044] Lu, F. , Lipka, A. E. , Glaubitz, J. , Elshire, R. , Cherney, J. H. , Casler, M. D. , Buckler, E. S. , & Costich, D. E. (2013). Switchgrass genomic diversity, ploidy, and evolution: Novel insights from a network‐based SNP discovery protocol. PLOS Genetics, 9, e1003215. 10.1371/journal.pgen.1003215 23349638 PMC3547862

[tpg220096-bib-0045] Moore, J. W. , Herrera‐Foessel, S. , Lan, C. , Schnippenkoetter, W. , Ayliffe, M. , Huerta‐Espino, J. , Lillemo, M. , Viccars, L. , Milne, R. , Periyannan, S. , Kong, X. , Spielmeyer, W. , Talbot, M. , Bariana, H. , Patrick, J. W. , Dodds, P. , Singh, R. , & Lagudah, E. (2015). A recently evolved hexose transporter variant confers resistance to multiple pathogens in wheat. Nature Genetics, 47, 1494. 10.1038/ng.3439 26551671

[tpg220096-bib-0046] Morrell, P. L. , & Clegg, M. T. (2007). Genetic evidence for a second domestication of barley (*Hordeum vulgare*) east of the Fertile Crescent. Proceedings of the National Academy of Sciences of the United States of America, 104, 3289. 17360640 10.1073/pnas.0611377104 PMC1805597

[tpg220096-bib-0047] Mujeeb‐Kazi, A. , Kazi, A. G. , Dundas, I. , Rasheed, A. , Ogbonnaya, F. , Kishii, M. , Bonnett, D. , Wang, R. R. C. , Xu, S. , Chen, P. D. , Mahmood, T. , Bux, H. , & Farrakh, S. (2013). Genetic diversity for wheat improvement as a conduit to food security. Advances in Agronomy, 122, 179–257. 10.1016/B978-0-12-417187-9.00004-8

[tpg220096-bib-0048] Narum, S. R. , & Hess, J. E. (2011). Comparison of FST outlier tests for SNP loci under selection. Molecular Ecology Resources, 11, 184–194. 10.1111/j.1755-0998.2011.02987.x 21429174

[tpg220096-bib-0049] Navarro, J. A. R. , Willcox, M. , Burgueño, J. , Romay, C. , Swarts, K. , Trachsel, S. , Preciado, E. , Terron, A. , Delgado, H. V. , Vidal, V. , Ortega, A. , Banda, A. E. , Montiel, N. O. G. , Ortiz‐Monasterio, I. , Vicente, F. S. , Espinoza, A. G. , Atlin, G. , Wenzl, P. , Hearne, S. , & Buckler, E. S. (2017). A study of allelic diversity underlying flowering‐time adaptation in maize landraces. Nature Genetics, 49, 476. 10.1038/ng.3784 28166212

[tpg220096-bib-0050] Parolo, S. , Lacroix, S. , Kaput, J. , & Scott‐Boyer, M. P. (2017). Ancestors’ dietary patterns and environments could drive positive selection in genes involved in micronutrient metabolism—The case of cofactor transporters. Genes & Nutrition, 12, 1–10. 10.1186/s12263-017-0579-x 29043008 PMC5628472

[tpg220096-bib-0051] Poland, J. A. , Brown, P. J. , Sorrells, M. E. , & Jannink, J.‐L. (2012). Development of high‐density genetic maps for barley and wheat using a novel two‐enzyme genotyping‐by‐sequencing approach. PLOS ONE, 7, e32253. 10.1371/journal.pone.0032253 22389690 PMC3289635

[tpg220096-bib-0052] Pritchard, J. , Wen, X. , & Falush, D. (2010). Documentation for structure software: Version 2.3. University of Chicago.

[tpg220096-bib-0053] Pritchard, J. K. , Stephens, M. , & Donnelly, P. (2000). Inference of population structure using multilocus genotype data. Genetics, 155, 945–959. 10835412 10.1093/genetics/155.2.945 PMC1461096

[tpg220096-bib-0054] Ralph, P. , & Coop, G. (2010). Parallel adaptation: One or nany waves of advance of an advantageous allele? Genetics, 186, 647. 10.1534/genetics.110.119594 20660645 PMC2954473

[tpg220096-bib-0055] Rasheed, A. , & Xia, X. (2019). From markers to genome‐based breeding in wheat. Theoretical and Applied Genetics, 132, 767–784. 10.1007/s00122-019-03286-4 30673804

[tpg220096-bib-0056] Rasheed, A. , Xia, X. , Mahmood, T. , Quraishi, U. M. , Aziz, A. , Brux, H. , Mahmood, Z. , Mirza, J. I. , Mujeeb‐Kazi, A. , & He, Z. (2016). Comparison of economically important loci in landraces and improved wheat cultivars from Pakistan. Crop Science, 56, 287–301. 10.2135/cropsci2015.01.0015

[tpg220096-bib-0057] Reif, J. C. , Zhang, P. , Dreisigacker, S. , Warburton, M. L. , van Ginkel, M. , Hoisington, D. , Bohn, M. , & Melchinger, A. E. (2005). Wheat genetic diversity trends during domestication and breeding. Theoretical and Applied Genetics, 110, 859–864. 10.1007/s00122-004-1881-8 15690175

[tpg220096-bib-0058] Ren, J. , Sun, D. , Chen, L. , You, F. M. , Wang, J. , Peng, Y. , Nevo, E. , Sun, D. , Luo, M. C. , & Peng, J. (2013). Genetic diversity revealed by single nucleotide polymorphism markers in a worldwide germplasm collection of durum wheat. International Journal of Molecular Sciences, 14, 7061–7088. 10.3390/ijms14047061 23538839 PMC3645677

[tpg220096-bib-0059] Riaz, A. , Athiyannan, N. , Periyannan, S. , Afanasenko, O. , Mitrofanova, O. , Aitken, E. A. B. , Lagudah, E. , & Hickey, L. T. (2016). Mining Vavilov's treasure chest of wheat diversity for adult plant resistance to *Puccinia triticina* . Plant Disease, 101, 317–323. 10.1094/PDIS-05-16-0614-RE 30681925

[tpg220096-bib-0060] Riaz, A. , Hathorn, A. , Dinglasan, E. , Ziems, L. , Richard, C. , Singh, D. , Mitrofanova, O. , Afanasenko, O. , Aitken, E. , Godwin, I. , & Hickey, L. (2017). Into the vault of the Vavilov wheats: Old diversity for new alleles. Genetic Resources and Crop Evolution, 64, 531–544. 10.1007/s10722-016-0380-5

[tpg220096-bib-0061] Russell, J. , Mascher, M. , Dawson, I. K. , Kyriakidis, S. , Calixto, C. , Freund, F. , Bayer, M. , Milne, I. , Marshall‐Griffiths, T. , Heinen, S. , Hofstad, A. , Sharma, R. , Himmelbach, A. , Knauft, M. , van Zonneveld, M. , Brown, J. W. S. , Schmid, K. , Kilian, B. , Muehlbauer, G. J. , … Waugh, R. (2016). Exome sequencing of geographically diverse barley landraces and wild relatives gives insights into environmental adaptation. Nature Genetics, 48, 1024–1030. 10.1038/ng.3612 27428750

[tpg220096-bib-0062] Saghai‐Maroof, M. A. , Soliman, K. M. , Jorgensen, R. A. , & Allard, R. (1984). Ribosomal DNA spacer‐length polymorphisms in barley: Mendelian inheritance, chromosomal location, and population dynamics. Proceedings of the National Academy of Sciences, 81, 8014–8018. 10.1073/pnas.81.24.8014 PMC392284 6096873

[tpg220096-bib-0063] Saitou, N. , & Nei, M. (1987). The neighbor‐joining method: A new method for reconstructing phylogenetic trees. Molecular Biology and Evolution, 4, 406–425. 3447015 10.1093/oxfordjournals.molbev.a040454

[tpg220096-bib-0064] Salamini, F. , Özkan, H. , Brandolini, A. , Schäfer‐Pregl, R. , & Martin, W. (2002). Genetics and geography of wild cereal domestication in the near east. Nature Reviews Genetics, 3, 429. 10.1038/nrg817 12042770

[tpg220096-bib-0065] Sehgal, D. , Vikram, P. , Sansaloni, C. P. , Ortiz, C. , Pierre, C. S. , Payne, T. , Ellis, M. , Amri, A. , Petroli, C. D. , Wenzl, P. , & Singh, S. (2015). Exploring and mobilizing the gene bank biodiversity for wheat improvement. PLOS ONOE, 10, e0132112. 10.1371/journal.pone.0132112 PMC4503568 26176697

[tpg220096-bib-0066] Vavilov, N. I. (1926). Studies on the origin of cultivated plants. Bulletin of Applied Botany and Plant Breeding, 14, 1–245.

[tpg220096-bib-0067] Vavilov, N. I. (1992). Origin and geography of cultivated plants. Cambridge University Press.

[tpg220096-bib-0068] Vikram, P. , Franco, J. , Burgueño‐Ferreira, J. , Li, H. , Sehgal, D. , Saint Pierre, C. , Ortiz, C. , Sneller, C. , Tattaris, M. , Guzman, C. , Sansaloni, C. P. , Fuentes‐Davila, G. , Reynolds, M. , Sonders, K. , Singh, P. , Payne, T. , Wenzl, P. , Sharma, A. , Bains, N. S. , … Singh, S. (2016). Unlocking the genetic diversity of Creole wheats. Scientific Reports, 6, 23092. 10.1038/srep23092 26976656 PMC4791556

[tpg220096-bib-0069] Wang, J. , Wen, W. , Hanif, M. , Xia, X. , Wang, H. , Liu, S. , Liu, J. , Yang, L. , Cao, S. , & He, Z. (2016). *TaELF3‐1DL*, a homolog of *ELF3*, is associated with heading date in bread wheat. Molecular Breeding, 36, 161. 10.1007/s11032-016-0585-5

[tpg220096-bib-0070] Webster, H. , Keeble, G. , Dell, B. , Fosu‐Nyarko, J. , Mukai, Y. , Moolhuijzen, P. , Bellgard, M. , Jia, J. Z. , Kong, X. Y. , Feuillet, C. , Choulet, F. , Appels, R. , & Consor, I. W. G. S. (2012). Genome‐level identification of cell wall invertase genes in wheat for the study of drought tolerance. Functional Plant Biology, 39, 569–579. 10.1071/FP12083 32480809

[tpg220096-bib-0071] Winfield, M. O. , Allen, A. M. , Burridge, A. J. , Barker, G. L. , Benbow, H. R. , Wilkinson, P. A. , Coghill, J. , Waterfall, C. , Davassi, A. , Scopes, G. , Pirani, A. , Webster, T. , Brew, F. , Bloor, C. , King, J. , West, C. , Griffiths, S. , King, I. , Bentley, A. R. , & Edwards, K. J. (2016). High‐density SNP genotyping array for hexaploid wheat and its secondary and tertiary gene pool. Plant Biotechnology Journal, 14, 1195–1206. 10.1111/pbi.12485 26466852 PMC4950041

[tpg220096-bib-0072] Winfield, M. O. , Allen, A. M. , Wilkinson, P. A. , Burridge, A. J. , Barker, G. L. A. , Coghill, J. , Waterfall, C. , Wingen, L. U. , Griffiths, S. , & Edwards, K. J. (2017). High density genotyping of the A.E. Watkins Collection of hexaploid landraces identifies a large molecular diversity compared to elite bread wheat. Plant Biotechnology Journal, 16, 165–175. 10.1111/pbi.12757 28500796 PMC5785351

[tpg220096-bib-0073] Yang, Z. B. , Bai, Z. Y. , Li, X. L. , Wang, P. , Wu, Q. X. , Yang, L. , Li, L. Q. , & Li, X. J. (2012). SNP identification and allelic‐specific PCR markers development for *TaGW2*, a gene linked to wheat kernel weight. Theoretical and Applied Genetics, 125, 1057–1068. 10.1007/s00122-012-1895-6 22643902

[tpg220096-bib-0074] Zhang, B. , Xu, W. , Liu, X. , Mao, X. , Li, A. , Wang, J. , Chang, X. , Zhang, X. , & Jing, R. (2017). Functional conservation and divergence among homoeologs of *TaSPL20* and *TaSPL21*, two SBP‐box genes governing yield‐related traits in hexaploid wheat. Plant Physiology, 174, 1177. 10.1104/pp.17.00113 28424214 PMC5462027

[tpg220096-bib-0075] Zhang, W. , Zhao, G. , Gao, L. F. , Kong, X. Y. , Guo, Z. , Wu, B. , & Jia, J. Z. (2016). Functional studies of heading date‐related gene *TaPRR73*, a paralog of *Ppd1* in common wheat. Frontiers in Plant Science 7, 772. 27313595 10.3389/fpls.2016.00772 PMC4887500

[tpg220096-bib-0076] Zhao, J. , Wang, Z. , Liu, H. , Zhao, J. , Li, T. , Hou, J. , Zhang, X. , & Hao, C. (2019). Global status of 47 major wheat loci controlling yield, quality, adaptation and stress resistance selected over the last century. BMC Plant Biology, 19, 1–14. 10.1186/s12870-018-1612-y 30606117 PMC6318892

[tpg220096-bib-0077] Zhou, Y. , Chen, Z. , Cheng, M. , Chen, J. , Zhu, T. , Wang, R. , Liu, Y. , Qi, P. , Chen, G. , Jiang, Q. , Wei, Y. , Luo, M.‐C. , Nevo, E. , Allaby, R. G. , Liu, D. , Wang, J. , Dvorák, J. , & Zheng, Y. (2018). Uncovering the dispersion history, adaptive evolution and selection of wheat in China. Plant Biotechnology Journal, 16, 280–291. 10.1111/pbi.12770 28635103 PMC5785339

[tpg220096-bib-0078] Zikhali, M. , Wingen, L. U. , & Griffiths, S. (2016). Delimitation of the *Earliness per se D1* (*Eps‐D1*) flowering gene to a subtelomeric chromosomal deletion in bread wheat (*Triticum aestivum*). Journal of Experimental Botany, 67, 287–299. 10.1093/jxb/erv458 26476691 PMC4682435

[tpg220096-bib-0079] Zikhali, M. , Wingen, L. U. , Leverington‐Waite, M. , Specel, S. , & Griffiths, S. (2017). The identification of new candidate genes Triticum aestivum FLOWERING LOCUS T3‐B1 (TaFT3‐B1) and TARGET OF EAT1 (TaTOE1‐B1) controlling the short‐day photoperiod response in bread wheat. Plant, Cell & Environment, 40, 2678–2690. 10.1111/pce.13018 PMC5669021 28667827

